# 10-Phenyl-10*H*-phenoxazine-4,6-diol tetra­hydro­furan monosolvate

**DOI:** 10.1107/S2414314620012766

**Published:** 2020-09-25

**Authors:** Aislinn C. Whalen, Claudia Hernandez Brito, Kyoung H. Choi, Ellen J. T. Warner, David A. Thole, Michael R. Gau, Patrick J. Carroll, Mitchell R. Anstey

**Affiliations:** a Department of Chemistry, Davidson College, Davidson, North Carolina, USA; b Department of Chemistry, University of Pennsylvania, Philadelphia, Pennsylvania, 19104-6323, USA; University of Kentucky, USA

**Keywords:** crystal structure, phenoxazine, tetra­hydro­furan, hydrogen bonding

## Abstract

In the crystalline state of the title solvate, C_18_H_13_NO_3_·C_4_H_8_O, hydrogen-bonding inter­actions between hydroxyl groups on a phenoxazine backbone and the tetra­hydro­furan solvent are observed that suggest the ability for this compound to act as a chelating ligand. The O⋯O donor–acceptor distances for this hydrogen bonding are 2.7729 (15) and 2.7447 (15) Å. The three-ring backbone of the phenoxazine bends out of planarity by 18.92 (3)°, as computed using mean planes that encompass each half of the three-ring structure, with the central N and O atoms forming the line of flexion. In the crystal, a π–π stacking arrangement exists between inversion-related mol­ecules, with a centroid-to-centroid distance of 3.6355 (11) Å. In the disordered tetra­hydro­furan solvate, all atoms except oxygen were modeled over two positions, with occupancies of 0.511 (8) and 0.489 (8).

## Abstract 2

In the crystalline state of the title solvate, C_18_H_13_NO_3_·C_4_H_8_O, hydrogen-bonding inter­actions between hydroxyl groups on a phenoxazine backbone and the tetra­hydro­furan solvent are observed that suggest the ability for this compound to act as a chelating ligand.

## Graphical abstract

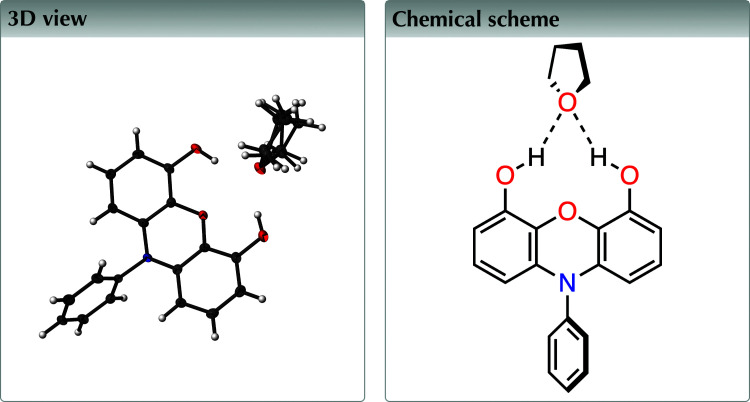

## Structure description

**Figure 1:**
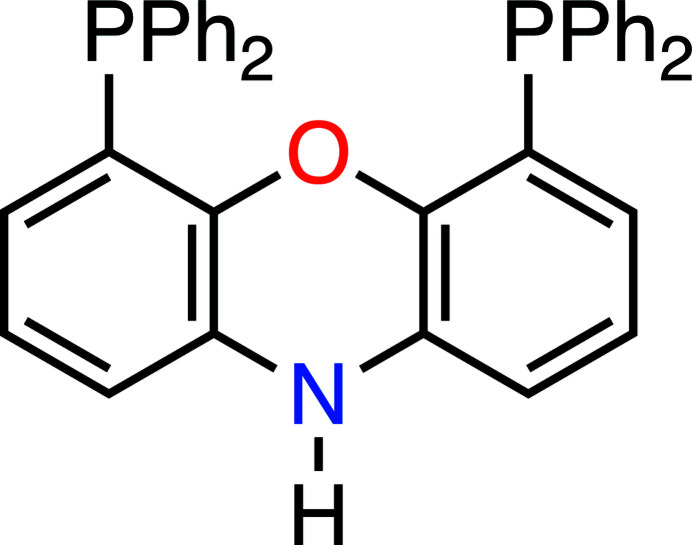
NiXantPhos ligand.

Phenoxazine-based metal complexes have been reported as catalysts in hydro­formyl­ation reactions (van der Veen *et al.*, 2000 ▸; Verheyen *et al.*, 2019 ▸), C—H bond aryl­ations (Li *et al.*, 2016 ▸), and aryl chloride cross-couplings (Zhang *et al.*, 2014 ▸). One of the most notable phenoxazine ligands is NiXantPhos (Fig. 1 ▸). The key to their utility lies in the ability of the ligand to chelate a metal center using the central oxygen atom (O1 in the reported case) alongside the functional groups at the 4 and 6 positions. As best as we can tell, there is not yet a report of a phenoxazine ligand with hydroxyl functional groups at these same positions offering the same ability to chelate.

**Figure 2:**
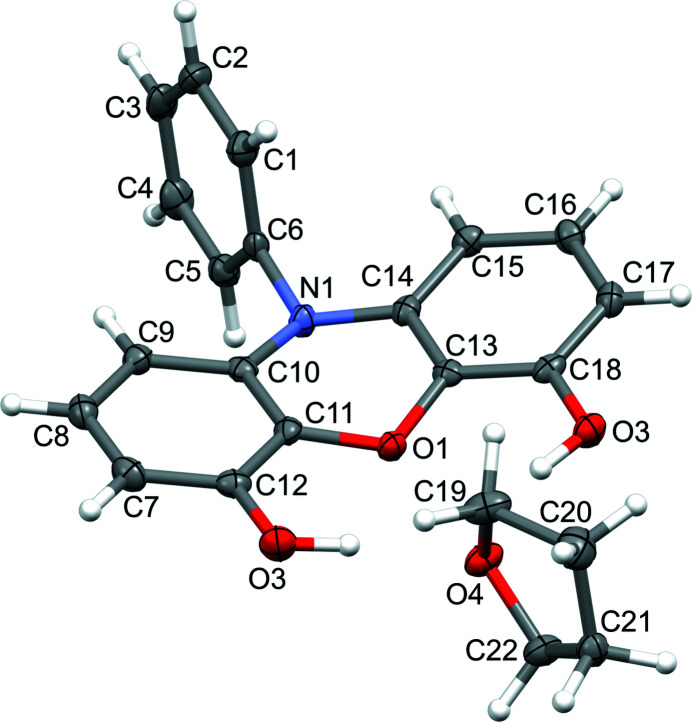
Ellipsoid plot (50%) of the title solvate. The minor component of disorder for the THF solvent mol­ecule is omitted for the sake of clarity.

The reported compound consists of a 10-phenyl-10*H*-phenoxazine backbone with two hydroxyl moieties at the 4 and 6 positions of the phenoxazine ring, and this structure was obtained as a tetra­hydro­furan solvate (Fig. 2 ▸). Similar to the other reported phenoxazine-based ligands, the phenoxazine fused ring system is not planar, with flexion at O1 and N1 resulting in an 18.92 (3)° deviation from planarity, as computed using mean planes that encompass each half of the three-ring structure (*i.e.*, atoms C7–C12/N1/O1 and C13–C18/N1/O1). The plane of the *N*-phenyl group is nearly perpendicular to the phenoxazine ring structure, with a dihedral angle of 89.14 (6)° between the mean plane of the phenyl ring and the plane defined by N1,C10,C11.

**Figure 3:**
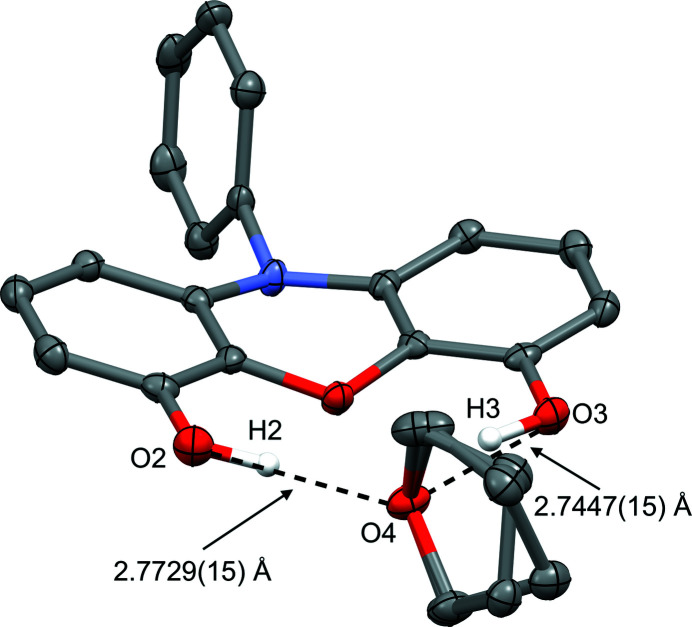
Solid-state structure of 10-phenyl-10*H*-phenoxazine-4,6-diol, with disordered tetra­hydro­furan solvate mol­ecule (50% ellipsoids). Hydrogen-bonding inter­actions between the two hydroxyl groups and the oxygen atom of tetra­hydro­furan are indicated by dashed lines.

This compound was crystallized from a solution of toluene and tetra­hydro­furan, and the resulting structure solution shows a single tetra­hydro­furan molecule with its oxygen atom accepting two hydrogen-bonding inter­action from the phenoxazine hydroxyl groups. The inter­actions between O4 in the tetra­hydro­furan solvent with the O2 and O3 hydroxyl groups mimic a structure that a deprotonated, dianionic form of the title compound might adopt upon complexation with a metal ion. The inter­action between these mol­ecules could be classified according to the Jeffrey model as ‘moderate, mostly electrostatic’ (Jeffrey, 1997 ▸) with donor–acceptor distances of 2.7729 (15) Å (O4⋯O2) and 2.7447 (15) Å (O4⋯O3) (Fig. 3 ▸, Table 1 ▸).

**Figure 4:**
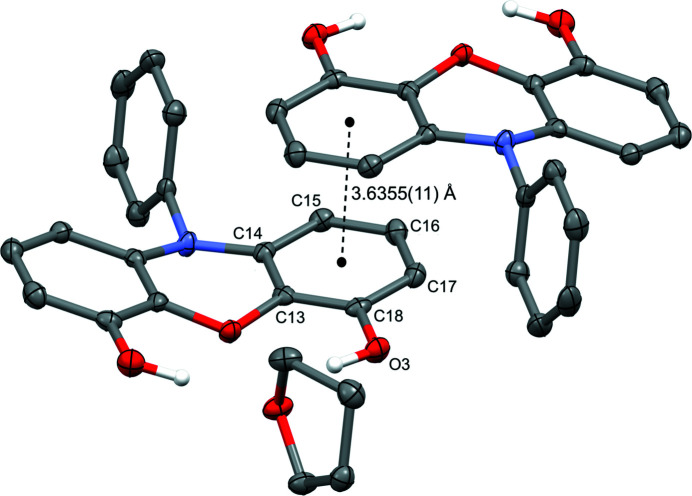
Solid-state structure of 10-phenyl-10*H*-phenoxazine-4,6-diol with the major THF component (50% ellipsoids). An additional inversion-related (1 − *x*, 1 − *y*, 1 − *z*) mol­ecule is included, showing the π–π stacking distance of 3.6355 (11) Å.

One other important supra­molecular feature is a π–π stacking inter­action between inversion-related (1 − *x*, 1 − *y*, 1 − *z*) mol­ecules (Fig. 4 ▸). One of the peripheral arene rings lies over the same ring in a neighboring mol­ecule. The centroid-to-centroid separation between these two rings is 3.6355 (11) Å.

## Synthesis and crystallization

10-Phenyl-10*H*-phenoxazine was synthesized according to literature procedures (Liu *et al.*, 2014 ▸). With this compound in hand, an anhydrous deprotonation was performed on 39 mmol of starting material using 2.2 equivalents of *n*-butyl­lithium (2.5 *M* in hexa­nes) and *N*,*N*,*N′*,*N′*-tetra­methyl­ethane-1,2-di­amine in diethyl ether solvent at 273 K. The solution was allowed to warm to room temperature and then stirred overnight. The reaction mixture of the li­thia­ted 10-phenyl-10*H*-phenoxazine was then cooled again to 273 K and cannulated into a stirred solution of diethyl ether and four equivalents of trimethyl borate. The solution was allowed to warm to room temperature and then stirred overnight. This initial procedure was modeled after the one reported for the synthesis of NiXantPhos (van der Veen *et al.*, 2009 ▸).

The lemon-yellow reaction mixture was evaporated on a rotary evaporator to dryness. The solids were redissolved in ∼500 ml of methanol and stirred until homogenous. A solution of methanol and six equivalents of urea hydrogen peroxide was prepared and then added dropwise at 273 K to the reaction mixture. The reaction darkened considerably, to a deep red. After stirring overnight, the reaction mixture was concentrated to one quarter of its initial volume using a rotary evaporator before being diluted with ∼400 ml of distilled water. The solution pH was adjusted using hydro­chloric acid until it was neutral to slightly acidic (pH 4–6 indicated by pH paper). At this point the reaction mixture contained a considerable amount of solid that was identified as the 10-phenyl-10*H*-phenoxazine-4,6-diol, so the reaction mixture was filtered to obtain this crude brown–red solid. The subsequent procedure is modeled after the hy­droxy­lation described by Gupta *et al.* (2016 ▸).

The compound was then purified using silica gel column chromatography with a solvent mixture of 15% ethyl acetate in hexa­nes as an eluent. The eluent polarity was increased by increasing the concentration of ethyl acetate up to 35% over the course of the procedure. The final compound was obtained in 50% yield.

Single crystals suitable for X-ray analysis were obtained using a vapor diffusion method. A small portion of the title compound was dissolved in tetra­hydro­furan and transferred to a small cylindrical vial that fitted fully into a standard 20 ml scintillation vial. The volume around the small vial was then filled with toluene until it reached approximately half the capacity of the remaining volume. The 20 ml vial was capped with the inter­nal vial uncapped to allow for vapors diffusion. In this embodiment, the tetra­hydro­furan solvent will evaporate and dissolve into the toluene solution, concentrating the title compound in the tetra­hydro­furan vial.

## Refinement

Crystal data, data collection and structure refinement details are summarized in Table 2 ▸. The positions of the two hydroxyl H atoms involved in hydrogen bonding, H2 and H3, were refined from difference-map peaks as proof of their correct assignment. The tetra­hydro­furan mol­ecule was found to be disordered, and all atoms except for oxygen were modeled across two positions. Due to the positioning of the disordered parts, *SHELXL* commands EADP and SAME were used to ensure a stable refinement. Disordered part occupancies refined to 0.511 (8) and 0.489 (8).

## Supplementary Material

Crystal structure: contains datablock(s) I. DOI: 10.1107/S2414314620012766/pk4031sup1.cif

Structure factors: contains datablock(s) I. DOI: 10.1107/S2414314620012766/pk4031Isup2.hkl

Supporting information file. DOI: 10.1107/S2414314620012766/pk4031Isup3.cml

CCDC reference: 2032769

Additional supporting information:  crystallographic information; 3D view; checkCIF report

## Figures and Tables

**Table 1:** Hydrogen-bond geometry (Å, °)

*D*—H⋯*A*	*D*—H	H⋯*A*	*D*⋯*A*	*D*—H⋯*A*
O2—H2⋯O4	0.93 (2)	1.88 (2)	2.7729 (15)	159.5 (19)
O3—H3⋯O4	0.91 (2)	1.88 (2)	2.7447 (15)	157.0 (19)

**Table 2:** Experimental details

Crystal data	
Chemical formula	C_18_H_13_NO_3_·C_4_H_8_O
*M*_r_	363.40
Crystal system, space group	Monoclinic, *P*2_1_/*n*
Temperature (K)	100
*a*, *b*, *c* (Å)	8.2694 (3), 21.1783 (8), 10.7050 (4)
β (°)	111.117 (1)
*V* (Å^3^)	1748.89 (11)
*Z*	4
Radiation type	Mo *K*α
μ (mm^−1^)	0.10
Crystal size (mm)	0.37 × 0.29 × 0.14

Data collection	
Diffractometer	Bruker D8 Quest CMOS Photon 100
Absorption correction	Multi-scan (*SADABS*; Bruker, 2018 ▸)
*T*_min_, *T*_max_	0.692, 0.745
No. of measured, independent and observed [*I* > 2σ(*I*)] reflections	27791, 3212, 2674
*R*_int_	0.046
(sin θ/λ)_max_ (Å^−1^)	0.604

Refinement	
*R*[*F* ^2^ > 2σ(*F* ^2^)], *wR*(*F* ^2^), *S*	0.037, 0.090, 1.06
No. of reflections	3212
No. of parameters	271
No. of restraints	76
H-atom treatment	H atoms treated by a mixture of independent and constrained refinement
Δρ_max_, Δρ_min_ (e Å^−3^)	0.17, −0.25

Computer programs: *SAINT* (Bruker, 2018 ▸), *SHELXT* (Sheldrick, 2015*a* ▸), *SHELXL* (Sheldrick, 2015*b* ▸) and *OLEX2* (Dolomanov *et al.*, 2009 ▸).

## full crystallographic data

### Crystal data

**Table:**

C_18_H_13_NO_3_·C_4_H_8_O	*F*(000) = 768
*M**_r_* = 363.40	*D*_x_ = 1.380 Mg m^−^^3^
Monoclinic, *P*2_1_/*n*	Mo *K*α radiation, λ = 0.71073 Å
*a* = 8.2694 (3) Å	Cell parameters from 9962 reflections
*b* = 21.1783 (8) Å	θ = 3.3–25.4°
*c* = 10.7050 (4) Å	µ = 0.10 mm^−^^1^
β = 111.117 (1)°	*T* = 100 K
*V* = 1748.89 (11) Å^3^	Plank, colourless
*Z* = 4	0.37 × 0.29 × 0.14 mm

### Data collection

**Table:**

Bruker D8 Quest CMOS Photon 100 diffractometer	2674 reflections with *I* > 2σ(*I*)
ω and φ scans	*R*_int_ = 0.046
Absorption correction: multi-scan (SADABS; Bruker, 2018)	θ_max_ = 25.4°, θ_min_ = 3.3°
*T*_min_ = 0.692, *T*_max_ = 0.745	*h* = −9→9
27791 measured reflections	*k* = −25→25
3212 independent reflections	*l* = −12→11

### Refinement

**Table:**

Refinement on *F*^2^	76 restraints
Least-squares matrix: full	Hydrogen site location: mixed
*R*[*F*^2^ > 2σ(*F*^2^)] = 0.037	H atoms treated by a mixture of independent and constrained refinement
*wR*(*F*^2^) = 0.090	*w* = 1/[σ^2^(*F*_o_^2^) + (0.0442*P*)^2^ + 0.5498*P*] where *P* = (*F*_o_^2^ + 2*F*_c_^2^)/3
*S* = 1.06	(Δ/σ)_max_ = 0.001
3212 reflections	Δρ_max_ = 0.17 e Å^−^^3^
271 parameters	Δρ_min_ = −0.25 e Å^−^^3^

### Special details

**Table:**

Geometry. All esds (except the esd in the dihedral angle between two l.s. planes) are estimated using the full covariance matrix. The cell esds are taken into account individually in the estimation of esds in distances, angles and torsion angles; correlations between esds in cell parameters are only used when they are defined by crystal symmetry. An approximate (isotropic) treatment of cell esds is used for estimating esds involving l.s. planes.

### Fractional atomic coordinates and isotropic or equivalent isotropic displacement parameters (Å^2^)

**Table:**

	*x*	*y*	*z*	*U*_iso_*/*U*_eq_	Occ. (<1)
O1	0.48901 (12)	0.35256 (4)	0.42474 (9)	0.0174 (2)	
O2	0.75882 (13)	0.27069 (5)	0.47249 (11)	0.0234 (2)	
H2	0.711 (3)	0.2971 (11)	0.399 (2)	0.057 (6)*	
O3	0.46637 (13)	0.44236 (5)	0.23825 (10)	0.0211 (2)	
H3	0.532 (3)	0.4065 (11)	0.259 (2)	0.056 (6)*	
N1	0.32048 (14)	0.36710 (5)	0.60817 (11)	0.0170 (3)	
C1	0.33959 (19)	0.42379 (7)	0.81239 (15)	0.0222 (3)	
H1	0.437572	0.446068	0.808879	0.027*	
C2	0.2799 (2)	0.43476 (7)	0.91599 (15)	0.0262 (4)	
H2A	0.337715	0.464365	0.984125	0.031*	
C3	0.1361 (2)	0.40269 (7)	0.92040 (16)	0.0271 (4)	
H3A	0.095770	0.410182	0.991689	0.033*	
C4	0.0514 (2)	0.35982 (7)	0.82124 (16)	0.0254 (3)	
H4	−0.047690	0.338086	0.824147	0.030*	
C5	0.11060 (18)	0.34832 (7)	0.71710 (15)	0.0205 (3)	
H5	0.052181	0.318918	0.648693	0.025*	
C6	0.25548 (18)	0.38013 (6)	0.71400 (14)	0.0166 (3)	
C7	0.73513 (18)	0.23963 (7)	0.67830 (15)	0.0216 (3)	
H7	0.826737	0.210208	0.692460	0.026*	
C8	0.65703 (18)	0.24613 (7)	0.77205 (15)	0.0206 (3)	
H8	0.697378	0.221394	0.851363	0.025*	
C9	0.52065 (17)	0.28807 (6)	0.75289 (14)	0.0173 (3)	
H9	0.467994	0.291674	0.818144	0.021*	
C10	0.46193 (17)	0.32481 (6)	0.63683 (13)	0.0154 (3)	
C11	0.54326 (17)	0.31868 (6)	0.54403 (13)	0.0155 (3)	
C12	0.67853 (17)	0.27654 (6)	0.56296 (14)	0.0178 (3)	
C13	0.39217 (16)	0.40665 (6)	0.42331 (13)	0.0148 (3)	
C14	0.30677 (17)	0.41490 (6)	0.51257 (13)	0.0155 (3)	
C15	0.20860 (17)	0.46948 (6)	0.50202 (14)	0.0175 (3)	
H15	0.149204	0.476407	0.562010	0.021*	
C16	0.19783 (17)	0.51370 (7)	0.40367 (14)	0.0193 (3)	
H16	0.130145	0.550674	0.396867	0.023*	
C17	0.28348 (17)	0.50509 (7)	0.31540 (14)	0.0190 (3)	
H17	0.274521	0.535752	0.248457	0.023*	
C18	0.38298 (17)	0.45100 (7)	0.32567 (13)	0.0162 (3)	
O4	0.69658 (12)	0.34718 (5)	0.24978 (10)	0.0227 (2)	
C19	0.8657 (14)	0.3770 (8)	0.3042 (10)	0.0246 (11)	0.511 (8)
H19A	0.957377	0.345769	0.348915	0.030*	0.511 (8)
H19B	0.867345	0.410702	0.368699	0.030*	0.511 (8)
C20	0.8879 (13)	0.4040 (5)	0.1804 (8)	0.0263 (13)	0.511 (8)
H20A	1.012157	0.408602	0.193679	0.032*	0.511 (8)
H20B	0.830823	0.445729	0.157328	0.032*	0.511 (8)
C21	0.8004 (6)	0.3555 (2)	0.0715 (4)	0.0213 (10)	0.511 (8)
H21A	0.749212	0.376122	−0.017070	0.026*	0.511 (8)
H21B	0.884354	0.323096	0.067003	0.026*	0.511 (8)
C22	0.660 (2)	0.3262 (7)	0.1139 (8)	0.0221 (4)	0.511 (8)
H22A	0.543625	0.340720	0.054752	0.026*	0.511 (8)
H22B	0.663826	0.279574	0.109859	0.026*	0.511 (8)
C19'	0.8568 (15)	0.3837 (9)	0.2936 (10)	0.0246 (11)	0.489 (8)
H19C	0.949284	0.359863	0.362841	0.030*	0.489 (8)
H19D	0.839036	0.424097	0.333275	0.030*	0.489 (8)
C20'	0.9104 (13)	0.3965 (6)	0.1747 (9)	0.0263 (13)	0.489 (8)
H20C	1.005949	0.368206	0.175162	0.032*	0.489 (8)
H20D	0.946960	0.440913	0.173706	0.032*	0.489 (8)
C21'	0.7445 (7)	0.3824 (3)	0.0560 (4)	0.0240 (11)	0.489 (8)
H21C	0.769927	0.368516	−0.023244	0.029*	0.489 (8)
H21D	0.666759	0.419599	0.031880	0.029*	0.489 (8)
C22'	0.667 (2)	0.3288 (7)	0.1124 (8)	0.0221 (4)	0.489 (8)
H22C	0.540968	0.324390	0.060592	0.026*	0.489 (8)
H22D	0.724585	0.288228	0.109658	0.026*	0.489 (8)

### Atomic displacement parameters (Å^2^)

**Table:**

	*U*^11^	*U*^22^	*U*^33^	*U*^12^	*U*^13^	*U*^23^
O1	0.0208 (5)	0.0185 (5)	0.0146 (5)	0.0032 (4)	0.0084 (4)	0.0009 (4)
O2	0.0243 (5)	0.0252 (6)	0.0250 (6)	0.0046 (4)	0.0141 (5)	0.0002 (5)
O3	0.0245 (5)	0.0250 (6)	0.0181 (5)	−0.0001 (4)	0.0127 (4)	0.0015 (4)
N1	0.0187 (6)	0.0189 (6)	0.0159 (6)	0.0034 (5)	0.0092 (5)	0.0036 (5)
C1	0.0257 (8)	0.0220 (7)	0.0207 (8)	−0.0019 (6)	0.0105 (6)	0.0024 (6)
C2	0.0413 (9)	0.0209 (8)	0.0187 (8)	0.0016 (7)	0.0134 (7)	−0.0003 (6)
C3	0.0411 (9)	0.0258 (8)	0.0239 (8)	0.0083 (7)	0.0231 (7)	0.0065 (7)
C4	0.0267 (8)	0.0256 (8)	0.0312 (9)	0.0026 (6)	0.0194 (7)	0.0071 (7)
C5	0.0225 (7)	0.0181 (7)	0.0226 (8)	0.0024 (6)	0.0103 (6)	0.0017 (6)
C6	0.0206 (7)	0.0158 (7)	0.0157 (7)	0.0046 (5)	0.0093 (6)	0.0041 (6)
C7	0.0184 (7)	0.0189 (7)	0.0269 (8)	0.0027 (6)	0.0075 (6)	0.0013 (6)
C8	0.0207 (7)	0.0184 (7)	0.0204 (7)	−0.0010 (6)	0.0047 (6)	0.0033 (6)
C9	0.0184 (7)	0.0180 (7)	0.0156 (7)	−0.0027 (5)	0.0064 (6)	−0.0009 (5)
C10	0.0148 (6)	0.0133 (6)	0.0166 (7)	−0.0024 (5)	0.0039 (5)	−0.0030 (5)
C11	0.0168 (7)	0.0150 (7)	0.0128 (7)	−0.0029 (5)	0.0030 (5)	−0.0004 (5)
C12	0.0168 (7)	0.0175 (7)	0.0204 (7)	−0.0036 (5)	0.0083 (6)	−0.0044 (6)
C13	0.0114 (6)	0.0155 (7)	0.0157 (7)	−0.0005 (5)	0.0025 (5)	−0.0018 (5)
C14	0.0135 (6)	0.0183 (7)	0.0135 (7)	−0.0032 (5)	0.0035 (5)	0.0001 (5)
C15	0.0150 (7)	0.0217 (7)	0.0167 (7)	0.0000 (5)	0.0069 (6)	−0.0005 (6)
C16	0.0165 (7)	0.0194 (7)	0.0214 (7)	0.0023 (5)	0.0061 (6)	0.0021 (6)
C17	0.0177 (7)	0.0204 (7)	0.0170 (7)	−0.0012 (6)	0.0040 (6)	0.0043 (6)
C18	0.0128 (6)	0.0223 (7)	0.0135 (7)	−0.0053 (5)	0.0049 (5)	−0.0032 (6)
O4	0.0216 (5)	0.0306 (6)	0.0188 (5)	−0.0030 (4)	0.0108 (4)	−0.0032 (4)
C19	0.0197 (11)	0.032 (3)	0.0227 (14)	−0.0022 (15)	0.0077 (10)	−0.0079 (15)
C20	0.026 (2)	0.024 (2)	0.0312 (10)	−0.0029 (17)	0.0131 (11)	0.0000 (10)
C21	0.022 (2)	0.024 (2)	0.0198 (16)	0.0008 (15)	0.0102 (15)	0.0017 (16)
C22	0.0231 (11)	0.0262 (11)	0.0187 (7)	−0.0033 (7)	0.0098 (6)	−0.0042 (7)
C19'	0.0197 (11)	0.032 (3)	0.0227 (14)	−0.0022 (15)	0.0077 (10)	−0.0079 (15)
C20'	0.026 (2)	0.024 (2)	0.0312 (10)	−0.0029 (17)	0.0131 (11)	0.0000 (10)
C21'	0.025 (2)	0.028 (2)	0.0222 (18)	−0.0051 (17)	0.0132 (16)	−0.0011 (17)
C22'	0.0231 (11)	0.0262 (11)	0.0187 (7)	−0.0033 (7)	0.0098 (6)	−0.0042 (7)

### Geometric parameters (Å, º)

**Table:**

O1—C11	1.3908 (16)	C15—H15	0.9500
O1—C13	1.3946 (16)	C15—C16	1.388 (2)
O2—H2	0.93 (2)	C16—H16	0.9500
O2—C12	1.3628 (17)	C16—C17	1.382 (2)
O3—H3	0.91 (2)	C17—H17	0.9500
O3—C18	1.3601 (16)	C17—C18	1.391 (2)
N1—C6	1.4448 (17)	O4—C19	1.451 (5)
N1—C10	1.4162 (17)	O4—C22	1.445 (5)
N1—C14	1.4147 (17)	O4—C19'	1.457 (5)
C1—H1	0.9500	O4—C22'	1.453 (5)
C1—C2	1.387 (2)	C19—H19A	0.9900
C1—C6	1.386 (2)	C19—H19B	0.9900
C2—H2A	0.9500	C19—C20	1.514 (6)
C2—C3	1.385 (2)	C20—H20A	0.9900
C3—H3A	0.9500	C20—H20B	0.9900
C3—C4	1.381 (2)	C20—C21	1.526 (7)
C4—H4	0.9500	C21—H21A	0.9900
C4—C5	1.391 (2)	C21—H21B	0.9900
C5—H5	0.9500	C21—C22	1.523 (7)
C5—C6	1.385 (2)	C22—H22A	0.9900
C7—H7	0.9500	C22—H22B	0.9900
C7—C8	1.382 (2)	C19'—H19C	0.9900
C7—C12	1.392 (2)	C19'—H19D	0.9900
C8—H8	0.9500	C19'—C20'	1.515 (6)
C8—C9	1.3916 (19)	C20'—H20C	0.9900
C9—H9	0.9500	C20'—H20D	0.9900
C9—C10	1.3965 (19)	C20'—C21'	1.526 (7)
C10—C11	1.3920 (19)	C21'—H21C	0.9900
C11—C12	1.3876 (19)	C21'—H21D	0.9900
C13—C14	1.3887 (19)	C21'—C22'	1.532 (8)
C13—C18	1.3868 (19)	C22'—H22C	0.9900
C14—C15	1.3936 (19)	C22'—H22D	0.9900
			
C11—O1—C13	115.33 (10)	C16—C17—C18	119.26 (13)
C12—O2—H2	112.7 (13)	C18—C17—H17	120.4
C18—O3—H3	110.4 (13)	O3—C18—C13	121.59 (13)
C10—N1—C6	117.55 (11)	O3—C18—C17	119.23 (12)
C14—N1—C6	118.36 (11)	C13—C18—C17	119.17 (13)
C14—N1—C10	117.00 (11)	C22—O4—C19	111.1 (6)
C2—C1—H1	120.2	C22'—O4—C19'	105.8 (6)
C6—C1—H1	120.2	O4—C19—H19A	111.3
C6—C1—C2	119.60 (14)	O4—C19—H19B	111.3
C1—C2—H2A	119.9	O4—C19—C20	102.5 (6)
C3—C2—C1	120.17 (14)	H19A—C19—H19B	109.2
C3—C2—H2A	119.9	C20—C19—H19A	111.3
C2—C3—H3A	120.0	C20—C19—H19B	111.3
C4—C3—C2	120.04 (14)	C19—C20—H20A	111.0
C4—C3—H3A	120.0	C19—C20—H20B	111.0
C3—C4—H4	119.9	C19—C20—C21	103.9 (8)
C3—C4—C5	120.24 (14)	H20A—C20—H20B	109.0
C5—C4—H4	119.9	C21—C20—H20A	111.0
C4—C5—H5	120.3	C21—C20—H20B	111.0
C6—C5—C4	119.42 (14)	C20—C21—H21A	110.9
C6—C5—H5	120.3	C20—C21—H21B	110.9
C1—C6—N1	119.79 (12)	H21A—C21—H21B	108.9
C5—C6—N1	119.68 (12)	C22—C21—C20	104.5 (5)
C5—C6—C1	120.52 (13)	C22—C21—H21A	110.9
C8—C7—H7	120.2	C22—C21—H21B	110.9
C8—C7—C12	119.50 (13)	O4—C22—C21	105.6 (4)
C12—C7—H7	120.2	O4—C22—H22A	110.6
C7—C8—H8	119.3	O4—C22—H22B	110.6
C7—C8—C9	121.50 (13)	C21—C22—H22A	110.6
C9—C8—H8	119.3	C21—C22—H22B	110.6
C8—C9—H9	120.3	H22A—C22—H22B	108.8
C8—C9—C10	119.36 (13)	O4—C19'—H19C	109.8
C10—C9—H9	120.3	O4—C19'—H19D	109.8
C9—C10—N1	122.71 (12)	O4—C19'—C20'	109.4 (7)
C11—C10—N1	118.56 (12)	H19C—C19'—H19D	108.2
C11—C10—C9	118.71 (12)	C20'—C19'—H19C	109.8
O1—C11—C10	121.88 (12)	C20'—C19'—H19D	109.8
C12—C11—O1	116.25 (12)	C19'—C20'—H20C	111.3
C12—C11—C10	121.82 (12)	C19'—C20'—H20D	111.3
O2—C12—C7	119.02 (12)	C19'—C20'—C21'	102.5 (7)
O2—C12—C11	121.89 (13)	H20C—C20'—H20D	109.2
C11—C12—C7	119.09 (13)	C21'—C20'—H20C	111.3
C14—C13—O1	121.88 (12)	C21'—C20'—H20D	111.3
C18—C13—O1	116.09 (12)	C20'—C21'—H21C	111.5
C18—C13—C14	122.01 (12)	C20'—C21'—H21D	111.5
C13—C14—N1	118.66 (12)	C20'—C21'—C22'	101.3 (7)
C13—C14—C15	118.31 (12)	H21C—C21'—H21D	109.3
C15—C14—N1	123.01 (12)	C22'—C21'—H21C	111.5
C14—C15—H15	120.1	C22'—C21'—H21D	111.5
C16—C15—C14	119.84 (13)	O4—C22'—C21'	104.9 (5)
C16—C15—H15	120.1	O4—C22'—H22C	110.8
C15—C16—H16	119.3	O4—C22'—H22D	110.8
C17—C16—C15	121.41 (13)	C21'—C22'—H22C	110.8
C17—C16—H16	119.3	C21'—C22'—H22D	110.8
C16—C17—H17	120.4	H22C—C22'—H22D	108.8
			
O1—C11—C12—O2	2.79 (18)	C10—N1—C14—C15	−160.96 (12)
O1—C11—C12—C7	−177.89 (12)	C10—C11—C12—O2	−179.69 (12)
O1—C13—C14—N1	0.62 (18)	C10—C11—C12—C7	−0.4 (2)
O1—C13—C14—C15	−178.17 (12)	C11—O1—C13—C14	−20.88 (17)
O1—C13—C18—O3	−1.08 (18)	C11—O1—C13—C18	160.53 (11)
O1—C13—C18—C17	177.75 (11)	C12—C7—C8—C9	1.1 (2)
N1—C10—C11—O1	−0.26 (19)	C13—O1—C11—C10	20.67 (17)
N1—C10—C11—C12	−177.64 (12)	C13—O1—C11—C12	−161.81 (11)
N1—C14—C15—C16	−178.48 (12)	C13—C14—C15—C16	0.25 (19)
C1—C2—C3—C4	−0.2 (2)	C14—N1—C6—C1	−68.80 (17)
C2—C1—C6—N1	−178.07 (12)	C14—N1—C6—C5	111.92 (14)
C2—C1—C6—C5	1.2 (2)	C14—N1—C10—C9	160.93 (12)
C2—C3—C4—C5	0.4 (2)	C14—N1—C10—C11	−20.49 (17)
C3—C4—C5—C6	0.2 (2)	C14—C13—C18—O3	−179.66 (12)
C4—C5—C6—N1	178.27 (12)	C14—C13—C18—C17	−0.84 (19)
C4—C5—C6—C1	−1.0 (2)	C14—C15—C16—C17	−0.3 (2)
C6—N1—C10—C9	10.81 (18)	C15—C16—C17—C18	−0.2 (2)
C6—N1—C10—C11	−170.60 (12)	C16—C17—C18—O3	179.60 (12)
C6—N1—C14—C13	170.18 (12)	C16—C17—C18—C13	0.7 (2)
C6—N1—C14—C15	−11.10 (19)	C18—C13—C14—N1	179.12 (12)
C6—C1—C2—C3	−0.6 (2)	C18—C13—C14—C15	0.34 (19)
C7—C8—C9—C10	−0.5 (2)	O4—C19—C20—C21	−35.2 (14)
C8—C7—C12—O2	178.66 (12)	O4—C19'—C20'—C21'	16.4 (16)
C8—C7—C12—C11	−0.7 (2)	C19—O4—C22—C21	−10.8 (16)
C8—C9—C10—N1	178.00 (12)	C19—C20—C21—C22	29.2 (14)
C8—C9—C10—C11	−0.58 (19)	C20—C21—C22—O4	−12.0 (14)
C9—C10—C11—O1	178.38 (11)	C22—O4—C19—C20	29.1 (15)
C9—C10—C11—C12	1.0 (2)	C19'—O4—C22'—C21'	−30.9 (15)
C10—N1—C6—C1	80.90 (16)	C19'—C20'—C21'—C22'	−33.6 (14)
C10—N1—C6—C5	−98.38 (15)	C20'—C21'—C22'—O4	40.6 (14)
C10—N1—C14—C13	20.31 (18)	C22'—O4—C19'—C20'	8.9 (17)

### Hydrogen-bond geometry (Å, º)

**Table:**

*D*—H···*A*	*D*—H	H···*A*	*D*···*A*	*D*—H···*A*
O2—H2···O4	0.93 (2)	1.88 (2)	2.7729 (15)	159.5 (19)
O3—H3···O4	0.91 (2)	1.88 (2)	2.7447 (15)	157.0 (19)

## References

[bb1] Bruker (2018). *APEX3*, *SAINT* and *SADABS*. Bruker AXS Inc., Madison, Wisconsin, USA.

[bb2] Dolomanov, O. V., Bourhis, L. J., Gildea, R. J., Howard, J. A. K. & Puschmann, H. (2009). *J. Appl. Cryst.* **42**, 339–341.

[bb3] Gupta, S., Chaudhary, P., Srivastava, V. & Kandasamy, J. (2016). *Tetrahedron Lett.* **57**, 2506–2510.

[bb4] Jeffrey, G. A. (1997). *An Introduction to Hydrogen Bonding*. Oxford University Press.

[bb5] Li, M., González-Esguevillas, M., Berritt, S., Yang, X., Bellomo, A. & Walsh, P. J. (2016). *Angew. Chem. Int. Ed.* **55**, 2825–2829. 10.1002/anie.201509757 PMC4887135 26846375

[bb6] Liu, N., Wang, B., Chen, W., Liu, C., Wang, X. & Hu, Y. (2014). *RSC Adv.* **4**, 51133–51139.

[bb7] Sheldrick, G. M. (2015*a*). *Acta Cryst.* A**71**, 3–8.

[bb8] Sheldrick, G. M. (2015*b*). *Acta Cryst.* C**71**, 3–8.

[bb9] Veen, L. A. van der, Keeven, P. H., Schoemaker, G. C., Reek, J. N. H., Kamer, P. C. J., van Leeuwen, P. W. N. M., Lutz, M. & Spek, A. L. (2000). *Organometallics*, **19**, 872–883.

[bb10] Verheyen, T., Santillo, N., Marinelli, D., Petricci, E., De Borggraeve, W. M., Vaccaro, L. & Smet, M. (2019). *ACS Appl. Polym. Mater.* **1**, 1496–1504.

[bb11] Zhang, J., Bellomo, A., Trongsiriwat, N., Jia, T., Carroll, P. J., Dreher, S. D., Tudge, M. T., Yin, H., Robinson, J. R., Schelter, E. J. & Walsh, P. J. (2014). *J. Am. Chem. Soc.* **136**, 6276–6287. 10.1021/ja411855d PMC4017615 24745758

